# Natural Infection by *Fasciola hepatica* in Red Deer (*Cervus elaphus*) from NW Spain: The Usefulness of Necropsy, Coprology, and Three Enzyme-Linked Immunosorbent Assays for the Diagnosis

**DOI:** 10.3390/ani15182649

**Published:** 2025-09-10

**Authors:** Sara González Hidalgo, Natividad Diez Baños, María del Rosario Hidalgo Argüello, Angelica Martínez-Delgado

**Affiliations:** 1 Department of Animal Health, Parasitology and Parasitic Diseases, Faculty of Veterinary Science, University of León, 24071 León, Spain; sgonzh00@estudiantes.unileon.es (S.G.H.); mndieb@unileon.es (N.D.B.); 2 Department of Biotechnology and Food Science, Faculty of Sciences, University of Burgos, 09001 Burgos, Spain; angelicamd@ubu.es

**Keywords:** red deer, *Fasciola hepatica*, natural infection, histopathological lesions, immunodiagnostic, Northwest Spain

## Abstract

The objective of this study was to investigate the epidemiology of *Fasciola hepatica* in *Cervus elaphus* kept in natural conditions in the Riaño Regional Hunting Reserve, north-west of Spain, where several species of domestic and wild animals coexist. One hundred red deer were examined and classified according to age, sex, and sampling season. After the necropsy of the animals, the liver was removed and inspected to obtain the adult parasites of *F. hepatica*. Faecal samples were collected and processed using the coprological sedimentation technique. The prevalence of this trematode by necropsy was 12%, with a low number of specimens per animal ($\bar{x}$ = 2.7 ± 1.5; range 1–6). The young animals and the males had a higher prevalence than the adults and the females, finding statistically significant differences only according to the host age. Significant variations were also observed when considering the seasons of the year, with the highest number of infected animals in spring. The histopathological study revealed the presence of lesions compatible with a chronic fasciolosis similar to that found in domestic animals. The shedding of *F. hepatica* eggs was quite low in terms of prevalence (6%) and mean intensity of infection ($\bar{x}$ = 27.3 ± 20.6, range 4–60 epg), being in young animals, in males, and in spring, where the greatest excretion of eggs was observed. These results suggest that the deer are suitable definitive hosts for *F. hepatica* in the northwest of the Iberian Peninsula, but they are unusual hosts. The serum samples were analysed using a native excretory/secretory antigen (FhES) and a 2.9 kDa recombinant protein (FhrAPS) used for diagnosis of early and current fasciolosis in livestock. A commercial kit for serodiagnosis of *F. hepatica* in sheep and cattle, based on a monoclonal antibody (BIO K 211), was also evaluated in red deer. The seroprevalence of seropositivity of *F. hepatica* by FhES-ELISA was 32%, by FhrAPS-ELISA 13%, and by BIO K 211, 9%. In the three serological tests, the seroprevalence obtained was higher in adult animals, in males, and in spring. In the three serological tests used to understand the epidemiology of *F. hepatica* in red deer, we have observed that the sensitivity was low, perhaps due to the use of an anti-bovine IgG1 as a conjugate, so in future immunodiagnostic tests, it would be more desirable to obtain an anti-deer IgG, probably achieving better results. Due to these results, it is essential to investigate other serological or molecular tests that allow us to know the real importance of *F. hepatica* in deer and other wild animals. The role of deer as a reservoir of this trematode does not appear to be very important.

## Simple Summary

The objective of this study was to investigate the epidemiology of *Fasciola hepatica* in *Cervus elaphus* kept in natural conditions in the Riaño Regional Hunting Reserve, north-western of Spain. One hundred red deer were examined and classified according to age, sex, and sampling season. After the necropsy of the animals, the liver was removed and inspected. Faecal samples were collected and processed using the coprological sedimentation technique. The prevalence of this trematode by necropsy was 12%, with a low number of specimens per animal ($\bar{x}$ = 2.7 ± 1.5; range 1–6). The young animals and the males had a higher prevalence with statistically significant differences only according to the host age. Significant variations were also observed when considering the seasons of the year, with the highest number of infected animals in spring. The histopathological study revealed the presence of lesions compatible with chronic fasciolosis. The shedding of *F. hepatica* eggs was quite low in terms of prevalence (6%) and mean intensity of infection ($\bar{x}$ = 27.3 ± 20.6, range 4–60). The samples were also analysed using excretory/secretory antigens (FhES) and a 2.9 kDa recombinant protein (FhrAPS) used for diagnosis of early and current fasciolosis in livestock. A commercial kit for serodiagnosis of *F. hepatica* in sheep and cattle, based on a monoclonal antibody (BIO K 211), was also evaluated in red deer. The prevalence of seropositivity of *F. hepatica* by FhES-ELISA was 32%, by FhrAPS-ELISA 13%, and by BIO K 211, 9%, being higher in adult animals, in males, and in spring.

## 1. Introduction

Fasciolosis is a parasitic disease caused by digenean trematodes belonging to the family Fasciolidae and the genus *Fasciola*. There are three described species, one of them only recorded in the hippopotamus in Zimbabwe, *F. nyanzae* [1]. The other two species, *F. hepatica* and *F. gigantica*, infect livestock and humans but have a different geographical distribution. *F. hepatica* is present in Europe, Asia, Africa, the Americas, and Oceania in areas where *Galba*/*Fossaria* lymnaeids are distributed, whereas *F. gigantica* appears restricted to Africa except the Maghreb and Asia except the high-altitude areas of the Himalayas. Its absence in other continents is being related to the lack of *Radix* species [2]. Both species cause a foodborne zoonotic disease with wild aquatic plants contaminated with metacercariae as a source of infection, and it was considered a secondary disease until the end of the 19th century [3]. Since then the situation has changed drastically, becoming an endemic or semi-endemic disease in different parts of the world. Different studies suggest that the number of human cases is increasing [4].

In our country, *F. hepatica* is the most important species affecting a wide range of hosts, including domestic ruminants (sheep, goats, cows), wild animals (deer, fallow deer, roe deer, Iberian ibex, chamois, mouflon, wild boar), donkeys, horses, rabbits, hares, and humans [5,6]. Its existence is linked to the presence of the semi-aquatic snail *Galba truncatula* as the main intermediate host [7,8]. The development of its life cycle in the environment includes a metacercarial stage that encysts on pasture and other vegetation and depends on geoclimatic, ecological, and anthropogenic factors such as altitude [9], temperature [10], precipitation, humidity, evapotranspiration [11], vegetation, and soil type [12]. Due to this, its spatial distribution is very uneven, and therefore there are large variations in prevalence [13].

The parasite causes liver damage, locating itself in the bile ducts and gallbladder of the animals. It occurs in three clinical forms—acute, subacute, and chronic—depending on the metacercariae ingested in a given time and the substantial liver damage caused by the migration of large numbers of juvenile fluke, with significant consequences for the health and welfare of the animal, causing great socio-economic impact. Acute infections can be fatal due to loss of liver function, with chronic infections that manifest themselves in anaemia, weight loss, and ill-thrift being more common [14,15,16].

Traditionally, *F. hepatica* infections are diagnosed by detecting eggs in faeces through coprological sedimentation or flotation techniques, but those techniques have a low sensitivity and can only detect patent infection [17]. In addition, it is difficult to establish a correlation between parasite burden and egg count in faeces. Even so, it remains a valuable diagnostic tool [18]. Infections can also be confirmed at necropsy. It is the safest and most effective method to be able to observe adult parasites and liver lesions.

Other methods for diagnosing include ELISAs testing for coproantigens and antibodies in serum and milk samples. These ELISAs are typically based on native fluke excretory/secretory (ES) products due to their strong immunogenic properties [19]. More recent testing methods have used several antigenic fractions of *Fasciola* spp., purified antigens and recombinant antigens, for the serodiagnosis of fasciolosis in human and animal species. Cysteine proteases, such as cathepsins, have proved to be good biomarkers of infection, and several tests have been developed to detect antibodies against this family of proteins, cathepsins L being the most used antigens [20,21,22]. Alternatively, animal fasciolosis can be diagnosed by immunological tests that detect coproantigens released by adult flukes living in bile ducts, as the ultrasensitive capture ELISA (MM3 COPRO) using the monoclonal antibody (mAb) MM3, and which is commercialised by Bio-X Diagnostics (La Jemelle, Belgium) [23,24,25]. Other diagnostic methods include molecular genetic techniques such as polymerase chain reaction (PCR) [26,27,28] or the loop-mediated isothermal amplification (LAMP) technique [29,30].

Although fasciolosis has been extensively studied in domestic animals and humans, the application of these methods in wild populations is necessary to elucidate the importance of *F. hepatica* in these hosts. However, it should be approached with caution because the course of the disease and previous health status with respect to other diseases are unknown [31].

The objectives of this study were (1) to investigate the epidemiology of *Fasciola hepatica* infection in red deer kept in natural conditions in a mountain area in the north of the province of León (Spain), (2) to know the effect of intrinsic factors, such as age, sex, or sampling seasons, on the *F. hepatica* infection, and (3) to evaluate and compare necropsy, coprology, and serological tests (excretory/secretory antigens, a recombinant *F. hepatica* surface antigen, and a commercial kit (BIO K 211) based on a monoclonal antibody) for fasciolosis in red deer under field conditions.

## 2. Materials and Methods

### 2.1. Study Area

**Figure 1:**
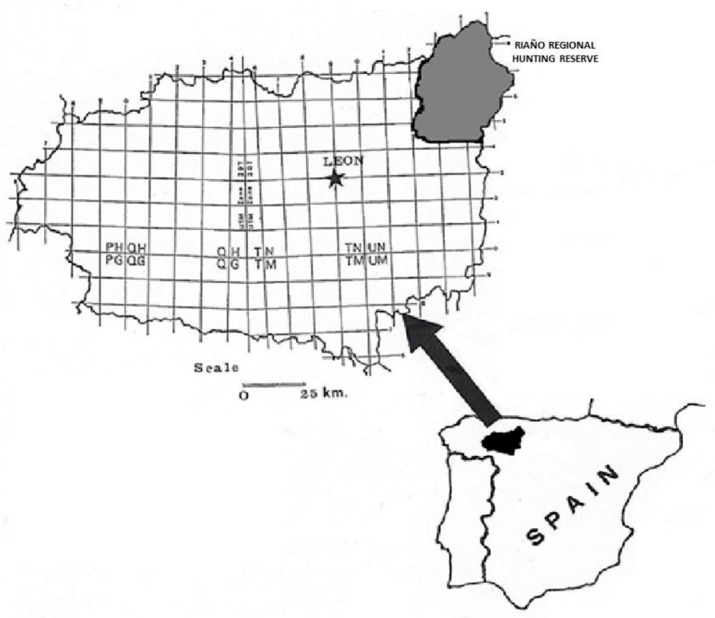
Riaño Regional hunting Reserve.

The present study was *carried out* in the Riaño Regional Hunting Reserve (43°03′14″ N, 4°57′33.85″ W), Province of León, north-western Spain, and belonging to the “Picos de Europa” National Park in the Cantabrian Mountains (Figure 1). The reserve is a protected area of 78,995 ha, and the greater part of its territory is in the Atlantic region, where the landscape is considerably mountainous, with altitudes between 1200 and 1500 m above sea level. The average monthly temperature is 10.2 °C (−12 °C/36 °C). Winter is long and cold, and summer is short and temperate with an average temperature of 16–17 °C. Rainfall occurs throughout the year, with an average annual precipitation of 1088.6 mm (maximum value in October, minimum in July), and snowfall occurs mainly between November and March [32]. The region has a wide and complex fluvial network, as well as numerous tributaries in the form of mountain streams.

The progressive abandonment of agricultural lands in this area has led to wild animals in the area (red deer, roe deer, chamois, wild boar, and Spanish ibex) sharing pastures and forests with domestic animals, mainly sheep and goats “farming in notable declive”, and cattle and horses raised for meat.

### 2.2. Animal Sampling

A total of 100 red deer were culled by the technical staff of the Reserve in selective hunting. For management reasons, it was not possible to obtain samples during the summer months. The animals sampled were classified according to their sex, age groups: young ≤ 2 years old and adults > 2 years old and sampling seasons (winter, spring and autumn), (Table 1).

### 2.3. Parasitological Examination

Once the animals were culled, and upon arrival at the faculty, they were identified and transferred to an enable room where a systematic and complete necropsy was performed. After accessing the abdominal cavity through the linea alba, the liver was extracted, first performing a macroscopic inspection of the entire surface of the organ. Next, the bile ducts and portal vein were opened, and the parenchyma was filleted into pieces about 1–3 cm thick. The pieces were pressed to facilitate the release of the trematodes, which were collected, washed with physiological solution, enumerated, and preserved in 70% ethanol at −20 °C for subsequent studies. The areas of the liver with representative lesions were preserved in 10% buffered formalin. After 48 h of fixation, they were paraffin-embedded, and tissue sections of 3 µm were stained with hematoxylin-eosin for histopathological study.

Blood samples were taken by cardiac puncture or from the thoracic cavity. Sera were obtained by centrifugation, placed in vials, labelled, and preserved at −20 °C until used. Also, faecal samples were collected directly from the rectum of the red deer and, after being labelled and identified, were immediately processed using the coprological sedimentation technique [33]. The samples were examined under a light microscope to detect and quantify *F. hepatica* eggs, which were expressed as the number of eggs per gram of faeces (epg).

### 2.4. Serological Procedures

Adult *F. hepatica* parasites collected from cattle livers at the slaughterhouse were washed several times in phosphate-buffered saline (0.1 mol/L PBS, pH 7.5) and finally incubated in RPMI culture medium [34,35]. Subsequently, the antigens obtained were dialyzed, lyophilized, and maintained as FhES (*F. hepatica*).

The presence of specific antibodies (IgG) against *F. hepatica* was investigated by enzyme-linked immunoassays (ELISA) using different antigens that had previously been tested in domestic ruminants experimentally and naturally infected [36,37]. The protocol was adapted to deer to detect the exposure against *F. hepatica*. The optimal concentrations of antigen, serum, and conjugate were previously determined by experiments using serum samples from deer that were positive and negative, by sedimentation, and by necropsy methods.

ELISA using excretory/secretory antigens of *F. hepatica* (FhES) was used at 1 μg ml^−1^ concentration, sera diluted (1/100) in PBS containing 0.05% Tween and 1% skimmed milk, and then anti-deer IgG peroxidase-labelled (Sigma Aldrich, St. Loius, MO, USA) was added and tested at 1/1000 [19].

Detection of IgG antibodies against the 2.9 kDa recombinant surface protein (APS) was performed in U-bottom microtiter plates coated with 3 μg ml^−1^ FhrAPS, sera diluted at 1/100, and anti-deer IgG peroxidase-labelled tested at 1/1000 [38,39].

The optical densities (OD) were read at 492 nm in an ELISA reader (Bio-Rad, Inc., Hercules, CA, USA, model 680 XR). Negative control sera were obtained from 5 young red deer that did not have flukes at necropsy and were negative for sedimentation. In order to establish the cut-off point, positive values were the mean optical density (OD) of negative sera plus 2 standard deviations (SD) [40,41]. Positive absorbance values were established in ≥0.1483 (FhES) and ≥0.1689 (FhrAPS).

Also, circulating IgG was investigated with a commercial test, “ELISA Kit for serodiagnosis of *F. hepatica* in bovine and ovine samples” (BIO K 211, Bio-X Diagnostics, La Jemelle, Belgium). This test uses as a conjugate a peroxidase-labelled anti-bovine IgG1 manufacturer and includes the interpretation of the results.

### 2.5. Statistical Analysis

The prevalence and mean intensity of the infection were determined considering host age, sex, and seasons of sampling [42]. The chi-square non-parametric test (χ^2^) was used to determine significant differences in prevalence, and the Mann–Whitney and Kruskal–Wallis tests were used to compare the intensity, taking different variables (host sex, age, and seasons) into account. The 95% confidence intervals (CI) for prevalence and mean intensity were calculated [43]. The sensitivity and specificity of the indirect ELISA were calculated according to the following formulas: sensitivity = true positive/true positive + false negative and specificity = true negative/false positive + true negative. The level of agreement among the ELISA methods, the necropsy, and/or coprology was assessed by the Kappa index (κ). Correlations between OD values and the number of flukes were assessed using Spearman’s rank correlation coefficient (ρ). Statistical significance was determined at the *p* ≤ 0.05 level. The statistical analysis was performed using the SPSS 18.0 software package (SPSS, Inc., Chicago, IL, USA).

## 3. Results

### 3.1. Necropsy

The prevalence of adult trematodes of *F. hepatica* obtained by necropsy was 12% (95% CI: 5.6–18.4%) with a mean intensity of 2.7 ± 1.6, 95% CI: 2.4–3, range 1–6 flukes/infected animal. The young animals had a higher prevalence (20%, 95% CI: 2.6–37.4%) than the adults (10%, 95% CI: 3.3–16.7), and the males (15.4%, 95% CI: 1.5–29.3%) had a higher prevalence than the females (10.8%, 95% CI: 3.8–17.8%), finding statistically significant differences only according to the host age (χ^2^ = 4.082, *p* = 0.043).

Significant variations were observed when considering the seasons of the year (χ^2^ = 6.270, *p* = 0.044), with the highest number of infected animals in spring (22.8%; 95% CI: 9–36) decreasing sharply in winter (7.9%, 95% CI: 1–16%) and autumn (3.7%, 95% CI: 1–11%). Regarding the mean intensity of infection, very low values were observed in all the parameters analysed, with no statistically significant differences found between them (Table 1).

**Figure 2:**
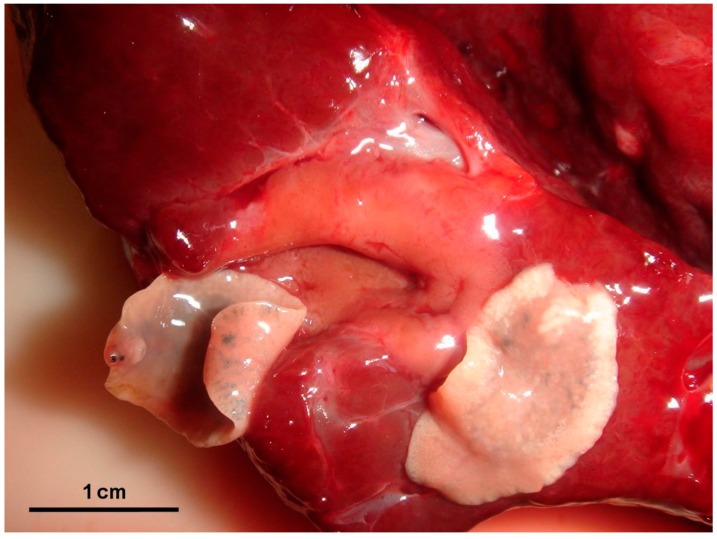
Large bile duct contained adults of *F. hepatica*. Note the thickened wall of the ecstatic bile duct.

**Figure 3:**
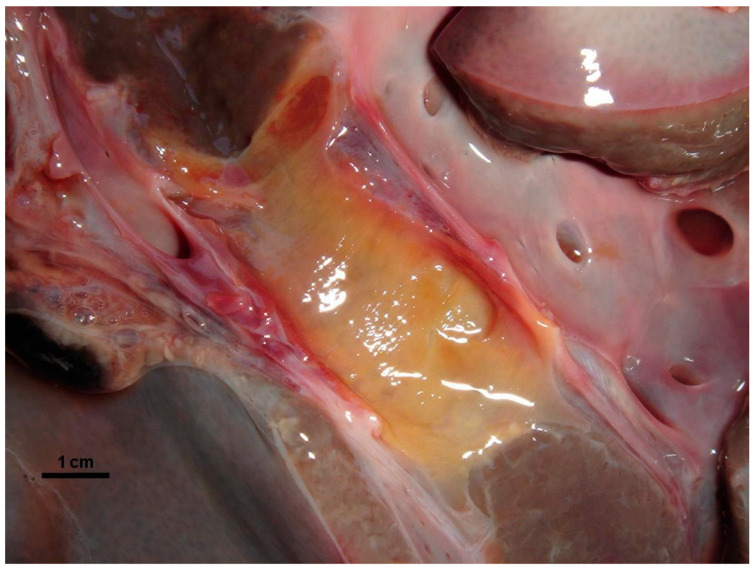
Severe epithelial hyperplasia and a blackish colour in the inner part of the hepatic lymph nodes.

**Figure 4:**
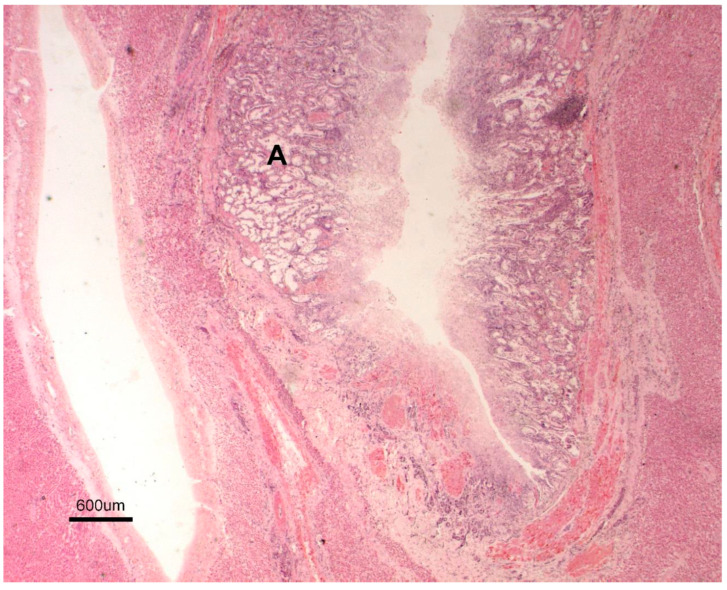
Large bile duct (H-E. 10×). A. Septal bile duct showing chronic cholangitis.

**Figure 5:**
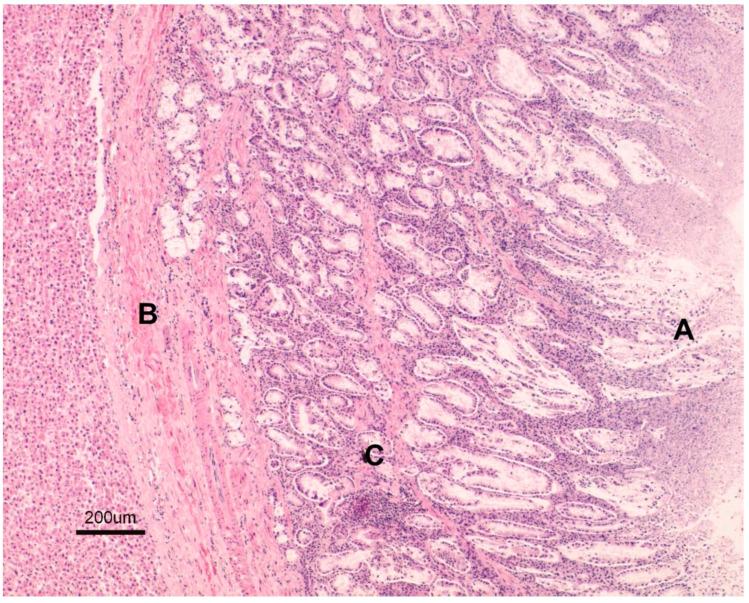
Detail of a large bile duct (H-E. 31×). A. Severe epithelial hyperplasia. B. Portal fibrosis. C. Severe inflammatory infiltrates.

Gross lesions were characterised by changes produced by the adult trematodes in the intrahepatic bile ducts, which had thickened walls and dilated lumens as they contained some parasites (Figure 2). Large areas of fibrosis were also observed in the liver parenchyma, calcification, and encapsulation of flukes. Furthermore, in the inner part of the hepatic lymph nodes, a blackish colour, associated with the presence of iron-porphyrin pigments, was observed (Figure 3). The main histopathological lesions were observed in the septal bile ducts and were characterised by chronic cholangitis and cholangiectasis (Figure 4). Affected bile ducts showed a moderate-severe epithelial hyperplasia with diffuse lymphocytic infiltration (Figure 5).

### 3.2. Coprology

The shedding of *F. hepatica* eggs was quite low in terms of prevalence (6%, 95% CI: 1.4–10.6) and mean intensity of infection ($\bar{x}$ = 27.3 ± 20.6, 95% CI: 23.2–31.4, range 4–60). Males (7.7%, 95% CI: 1–17.9%) and young animals (10%, 95% CI: 1–23.1%) had the highest prevalence, while females ($\bar{x}$ = 28.5 ± 23.6, 95% CI: 23.1–33.9) and adult animals ($\bar{x}$ = 32.5 ± 22.2; 95% CI: 27.6–37.4) had the highest mean intensity of infection. Regarding the seasons of the year, the highest prevalence and mean intensity of infection were observed in spring, with significant differences when comparing with the autumn (χ^2^ = 1.27, *p* = 0.053), but not with the winter values (*p* ≥ 0.05) (Table 1).

We must point out that only three deer harboured adult worms and excreted eggs in their faeces.

### 3.3. Serology

The prevalence of seropositivity of *F. hepatica* by FhES-ELISA was 32% (95% CI: 23–41%), by FhrAPS-ELISA 13% (95% CI: 6–20%), and by BIO K 211, 9% (95% CI: 3–15%). In the three serological tests, the seroprevalence obtained was higher in adult animals (11.3–36.3%) than in young (0–15%) and in males (15.4–42.3%) than in females (6.9–28.4%). Only significant differences were observed between adult and young animals tested with the FhES-ELISA (*p* ≤ 0.05). If we consider seasonal sampling, with the three tests analysed, the seroprevalence was higher in spring than in the rest of the seasons, but no statistically significant differences were found (Table 1).

**Figure 6:**
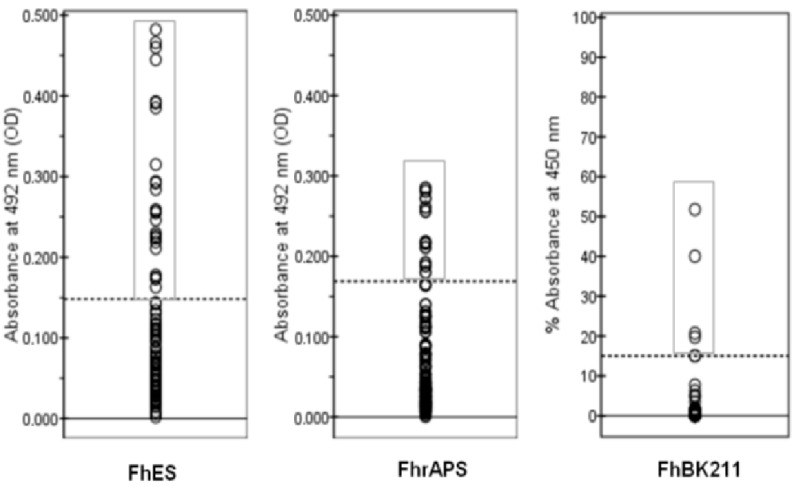
Distribution of OD values obtained with the FhES-ELISA (excretory-secretory products), FhrAPS-ELISA (2.9 kDa recombinant protein), and the FhBK211 (commercial version, BIO K 211, of the monoclonal antibody, MM3) in 88 serum samples from red deer negative to the “gold standard” (necropsy). Each circle represents the OD value for each animal tested with the 3 methods. The dashed lines represent cut-off values of the iELISA (≥0.1689 for FhrAPS, ≥0.1483 for FhES, and >15% absorbance for FhBK211). Circles inside the square represent false positive results.

When comparing the results between the three methods evaluated and the necropsy, it was observed that the highest number of false positives was detected with the FhES-ELISA (*n* = 25), and the highest number of false negatives was observed by both FhrAPS-ELISA (*n* = 10) and BIO K 211 (*n* = 9) methods (Figure 6). Five animals presented flukes in the liver and were negative by all three ELISA methods. The number of flukes correlated significantly with OD values only in the case of BIO K 211-ELISA (ρ = 0.675; *p* = 0.016).

Regarding the results obtained for sensitivity (Table 2), it was observed that its highest value was obtained for the FhES-ELISA (58.33%), followed by the BIO K 211-ELISA (25%) and the FhrAPS-ELISA (16.67%). The highest specificity and positive likelihood ratio (PLR) were obtained with the BIO K 211-ELISA. The concordance among the necropsy and the three ELISA methods evaluated was low: κ = 0.040 for FhrAPS-ELISA, κ = 0.174 for FhES-ELISA, and κ = 0.184 for BIO K 211-ELISA. The highest level of agreement was obtained between the FhES-ELISA and the BIO K 211-ELISA (κ = 0.326, *p* < 0.001).

The results obtained when comparing the three ELISAs studied and the coprology were also analysed. In the results obtained for sensitivity, it was observed that its highest value was obtained for the FhES-ELISA (66.67%), followed by the BIO K 211-ELISA (33.33%) and the FhrAPS-ELISA (16.67%); however, the specificity was much higher with the BIO K 211-ELISA than with the rest of the ELISAs analysed. The concordance between the three ELISAs and the coprology was very low, as can be seen from the results of the Kappa index (κ) (Table 3).

## 4. Discussion

The present study has detected the presence of *F. hepatica* in deer in a mountainous area in northern Spain where wild animals share their habitat with domestic livestock in a traditional free-range grazing system. Its knowledge is a fact of general biological interest from the veterinary and public health point of view.

The prevalence and intensity of infection found in this work has been low, both that obtained through necropsy (12%, $\bar{x}$ = 2.7 ± 1.5 flukes/infected animal) and that of the eggs faecal counts (6%, $\bar{x}$ = 27.3 ± 20.6 epg), although considerably higher than that observed in previous studies on this host: 7.27% flukes by necropsy, 3.64% eggs by coprology in this same study area [44], 1.64% adult flukes, 1.19% egg count in the central area of Spain [45], or 7% adult flukes in the south [19]. They were also very different from those found in other European deer populations, ranging between 0% in Germany [46], 7% in Ireland [47], 27.7% (flukes), 12.3% (faeces) in Scotland [31], or 31.3% in Belarus [48]. These large variations observed in the prevalence of infection are mainly due to environmental factors (topography, geochemistry, climate, and land use) that directly intervene in the development of the biological cycle of *F. hepatica* and the risk of infection, so they will be different in each of the study areas [49]. In addition to these factors, we must take into account susceptibility to infection in the different species of wildlife hosts. Thus, in our country, a low prevalence, 1.87% flukes and 0.53% eggs, was found in Iberian ibex in the Sierra Nevada mountains [5]; in roe deer, 1.87% flukes and 0.25% eggs; and in chamois, 0% flukes 0.39% eggs in our study area [50]; in 2% of fallow deer sampled in the south [19]; and in 6.1% of chamois in the Pyrenees [51]. These data contrast with the significant increase in prevalence obtained in domestic livestock since the last decade of the 19th century [52,53,54,55].

With both techniques we observed that the prevalence was higher in young animals and in males, while adult animals and females presented the highest intensity of infection. This same pattern has been observed in other parasitisms studied in wild animals in the same sampling area, where age would be related to the development of the hosts’ immune system, sex with the hormonal differences between them, different feeding habitats, land use, or physiological variations [56,57]. Regarding the seasons, the highest prevalence and intensity of infection were observed in spring, which correlates with the chronobiological pattern of *F. hepatica* in temperate areas [58,59].

The presence of trematodes in the inspection of the liver is 100% specific but does not always present the same sensitivity. This may be due to the pathogenesis caused by the parasite, with the formation of large areas of fibrosis in the liver parenchyma and bile duct walls. This results in significant calcification, leading to encapsulation of the flukes, preventing their migration through the bile ducts. This would result in fewer flukes reaching the bile ducts, preventing their sexual maturity; fewer adults detected at necropsy; and fewer eggs excreted in the faeces. This fact has been reported by various authors in deer and fallow deer [31,60]. The lesions found in the liver of this host are consistent with chronic fasciolosis described in domestic ruminants and other species [61,62,63]. Like necropsy, the coprological sedimentation technique for egg counting is highly specific but has low sensitivity. In addition to the above mentioned, this technique depends on a wide variety of factors, such as the faecal production of the animal, the volume of the sample analysed, the number of adult parasites present in the host, or the migration of immature stages and therefore the non-elimination of eggs. Furthermore, it must be taken into account that there are hourly, daily, and weekly variations in the elimination of eggs, especially in mild infections such as in deer [64,65,66].

When analysing both methods together, we found great disparity in results, and there is no correlation between the number of adults found and the egg count in the faeces. Nor is it known the number of eggs that adult parasites can eliminate or the longevity of *F. hepatica* in its hosts. However, from an epidemiological point of view, the use, especially of the coprological technique, is effective in knowing the parasitological status of certain hosts in a given area and is the technique used initially in the diagnosis of any parasitic process.

The data obtained in this study confirm that red deer, like other wild animals, are suitable definitive hosts for *F. hepatica* (they can complete their biological cycle), but they are unusual hosts (low rates of infection and partially inhibited development) [56,67]. For this reason, some authors state that their role as reservoirs can be reduced and mediated by ecological barriers that limit exposure and/or inherent resistance to infection [51,68], while others maintain that red deer could have an important role in the spreading of *F. hepatica* in areas shared with domestic ruminants [69].

Immunological techniques have been used successfully in the diagnosis of fasciolosis due to their high sensitivity, but studies of these techniques and their comparison with necropsy or the shedding of eggs in faeces are scarce. This study evaluates three ELISA methods for the detection of IgG antibodies in serum samples for the diagnosis of *F. hepatica* infection in red deer using excretory/secretory antigens, a recombinant *F. hepatica* surface antigen, and a commercial kit (BIO K 211) based on a monoclonal antibody. Many of these techniques have been tested in experimental infections in domestic animals, but it has been proven that the sensitivity and specificity of several of these methods decrease considerably under field conditions [36,38,65], and the values observed in the present study agree with these observations.

Thus, the 2.9 kDa recombinant protein had a sensitivity of 16.67% in the present work, while its values in sheep under experimental conditions were considerably higher (91%) [70], but these values decrease considerably (67.7%) in sheep naturally exposed to *F. hepatica* [36]. However, the sensitivity and specificity of the ELISA based on excretory/secretory antigens, 58% and 72% in the present study, respectively, showed higher values than those from sheep under field conditions (52.5%, 47.8%) but lower than those from sheep experimentally infected (86%, 97.5%) [36]. The monoclonal antibody is 100% sensitive and 100% specific for detecting early and long-term fasciolosis in sheep [24,71], values much higher than those found in this study.

Considering wild ruminants, the only comparable study was performed in Spain [17,39], analysing for the first time the presence of antibodies against excretory/secretory antigens of *F. hepatica* in these hosts. Our results were similar to those found in the red deer (34%), lower than those found in the fallow deer (58%), but higher than those observed in mouflon (12%), Iberian ibex (15%) from Southern Spain, and roe deer (25%) from Galicia. This is probably because red deer and fallow deer have been found to be more suitable hosts to *F. hepatica* infections than other wild ruminants due to their ecology and behaviour, despite controversy about their role in the epidemiology of this parasite.

Regarding factors such as age or sex, unlike what was observed in the necropsy and in the shedding of eggs in faeces, the seroprevalence was higher in adult animals and in males, which would be related mainly to ecological and physiological differences between them [57,72].

Several false positive results have been observed with the FhES-ELISA, and this seems to indicate the possibility of cross-reactions with antigens present in faeces common to other trematodes such as *Dicrocoelium dendriticum* and Paramphistomidae parasites [73,74,75,76]. On the other hand, some of these false positive results could correspond to primo-infected animals with juvenile flukes migrating through the intestinal wall and peritoneal cavity or animals that were previously exposed to the parasite but in which the infection has not been successfully established since the presence of antibodies do not always correlate with the existence of active infection but only with exposure to the parasite [77,78].

Also, the number of false negatives was higher with the recombinant protein and the monoclonal antibody than with the excretory/secretory antigens, despite the fact that the first two antigens are highly sensitive to detecting current and long-term sheep fasciolosis, even in animals infected with low numbers of flukes [70,71], or the discovery of five animals that harboured flukes and that were negative to the three assays, suggesting that other causes may be impairing the normal functioning of the immune system in these animals.

## 5. Conclusions

The present study has detected the presence of *F. hepatica* in deer in a mountainous area in northern Spain where wild animals share their habitat with domestic livestock in the area in a traditional free-range grazing system.

Regarding the diagnosis, we have observed that there was no correlation between the coprological technique and the necropsy findings, while the serological tests showed great variability of results both in prevalence and in sensitivity and specificity, so it would be essential to investigate other serological or molecular tests that allow us to know the real importance of *F. hepatica* in deer and other wild animals.

The ecological conditions of this area favour the natural development of the *F. hepatica* cycle, and it is confirmed that the deer are suitable definitive hosts for this trematode. However, the low results obtained in terms of prevalence and intensity of infection, number of eggs excreted in faeces and seroprevalence seem to indicate that the pastures shared by domestic and wild ruminants are little contaminated with the infective phase of *Fasciola hepatica* from deer, and their role as reservoir and transmission to domestic livestock does not appear to be very important. It is not known whether other wild animals could act as more suitable hosts for maintaining the disease.

## Figures and Tables

**Table 1:** Prevalence (%) and mean intensity (±SD) of infection by *F. hepatica* and percentage of animals by iELISA using FhES, FhrAPS and BIO K 211 relative to host age, sex, and seasons of sampling in red deer from NW Spain.

	Host Age		Sex		Seasons			Total *n* = 100
	≤2 Years *n* = 20	>2 Years *n* = 80	Males *n* = 26	Females *n* = 74	Autum *n* = 27	Winter *n* = 38	Spring *n* = 35	
Necropsy %	20	10	15.4	10.8	3.7	7.9	22.8	12
$\bar{x}$ ± SD	2.7 ± 1.7	2.6 ± 1.6	1.7 ± 0.9	3.1 ± 1.6	1	1.7 ± 1.5	3.2 ± 1.5	2.7 ± 1.5
Range	1–5	1–6	1–3	1–6	1	1–3	2–6	1–6
Egg output (epg) %	10	5	7.7	5.4	7.4	2.6	8.6	6
$\bar{x}$ ± SD	17 ± 18.4	32.5 ± 22.2	25 ± 21.2	28.5 ± 23.6	20 ± 14.1	40	28 ± 28.8	27.3 ± 20.6
Range	4–30	10–60	10–40	4–60	10–30	40	4–60	4–60
FhES-ELISA %	15	36.3	42.3	28.4	37	23.7	37.1	32
FhrAPS-ELISA %	5	15	23.1	9.5	3.7	13.2	20	13
BIO K 211-ELISA %	0	11.3	15.4	6.9	11.1	5.3	11.4	9

**Table 2:** Statistical analysis of the results obtained between the ELISAs studied and the necropsy.

	FhES-ELISA	FhrAPS-ELISA	BIO K 211-ELISA
Infected animals (*n* = 12)	7	2	3
Uninfected animals (*n* = 88)	63	77	82
Sensitivity %	58.33	16.67	25
Specificity %	71.60	87.50	93.18
PPV %	21.88	15.38	33.33
NPV %	92.65	88.51	90.11
PLR	2.053	1.333	3.700
NLR	0.582	0.952	0.805
κ-value	0.174	0.040	0.184
*p*-value	0.037	0.687	0.065
Seroprevalence (95% CI)	32 (23–41)	13 (6–20)	9 (3–15)

PPV = positive predictive value; NPV= negative predictive value; PLR = positive likelihood ratio; NLR = negative likelihood ratio.

**Table 3:** Statistical analysis of the results obtained between the ELISAs studied and the 40 coprology.

	FhES-ELISA	FhrAPS-ELISA	BIO K 211-ELISA
Infected animals (*n* = 6)	4	1	2
Uninfected animals (*n* = 94)	66	82	87
Specificity %	66.67	16.67	33.33
Specificity %	70.21	87.23	92.55
PPV %	12.50	3.12	6.25
NPV %	97.06	92.64	94.12
PLR	2.238	1.305	4.474
NLR	0.475	0.955	0.720
κ-value	0.122	−0.054	0.005
Seroprevalence (95% CI)	32 (23–41)	13 (6–20)	9 (3–15)

PPV = positive predictive value; NPV= negative predictive value; PLR = positive likelihood ratio; NLR = negative likelihood ratio.

## Data Availability

The data that support the findings of this study are included in the article.
